# Microwave Pretreatment of Soybeans Prior to Soaking Enhances Mechanical and Rehydration Properties of Yuba

**DOI:** 10.3390/foods15061094

**Published:** 2026-03-20

**Authors:** Weiyu Li, Siyu Zhan, Ke Sun, Chunli Song, Jian Ren

**Affiliations:** 1 College of Food and Bioengineering, Qiqihar University, Qiqihar 161006, China; 17604520971@163.com (W.L.); zsy1990882611@163.com (S.Z.); 03941@qqhru.edu.cn (K.S.); 2 Engineering Research Center of Plant Food Processing Technology, Ministry of Education, Qiqihar 161006, China

**Keywords:** microwave, yuba, presoaking, soymilk, mechanical properties

## Abstract

Microwave pretreatment of native soybeans in the preparation of yuba remains underexplored, and the impact of this treatment on the resulting yuba quality is still unclear. In this study, soybeans were subjected to microwave pretreatment for 30–120 s before conventional soaking. CLSM revealed soybean microstructural changes, including cell-wall degradation and improved dispersion of proteins and lipids. FTIR and SDS-PAGE results of yuba indicated that hydrogen bond cleavage and the formation of new cross-links reduced protein coiling and polar group exposure, while stabilizing aliphatic chains, ultimately yielding a stronger and more compact yuba network structure. Mechanical and rehydration results further indicated that microwave treatment positively affected yuba quality. The 90 s pretreatment was identified as the optimal condition, exhibiting the highest elongation at break (126.36% increase) and rehydration capacity, along with improved color attributes, including higher lightness (L*) and yellowness (b*) values. These changes are likely attributable to disulfide-mediated protein reorganization, which creates greater spatial availability and thereby facilitates lipid incorporation. This study elucidates how microwave pretreatment drives the reorganization of soybean protein and lipid components, thereby influencing their distribution during film formation and providing a foundation for the tailored design of yuba with targeted mechanical properties.

## 1. Introduction

Yuba is a protein-lipid self-assembled composite film formed at the soymilk-air interface during heating, widely valued for its high nutritional content and meat-like texture [1]. The film formation process relies on the complex composition of soymilk, which includes oil bodies, protein aggregates, soluble proteins, and carbohydrates. Upon heating, proteins in soymilk undergo thermal denaturation and form intermolecular disulfide bonds via oxidation, leading to the development of a three-dimensional network structure. Simultaneously, dispersed components migrate to the air-water interface, where amphiphilic proteins reorient with hydrophobic domains facing the air, and hydrophilic regions toward the aqueous phase. Oil bodies become entrapped within the evolving matrix, and continuous water evaporation further densifies the structure, ultimately generating the hierarchical porous architecture characteristic of yuba [2,3]. Consequently, the initial conformation of proteins and the dispersion state of oil bodies critically influence interfacial assembly behavior, thereby determining the microstructure, mechanical properties, and rehydration behavior of the final film [3].

Current strategies to enhance yuba quality focus on chemical and enzymatic modification of soybean proteins. Acidification regulates soymilk physicochemical properties and protein aggregation, thereby affecting film formation and structural integrity [4], while transglutaminase-assisted crosslinking strengthens intermolecular interactions and network stability, leading to improved tensile strength and water barrier properties [5]. Together, these approaches highlight the importance of protein conformational and interactional regulation in optimizing yuba quality. However, increasing attention has been directed toward physical field technologies as green and efficient alternatives.

Microwave technology serves as an efficient physical modification method, operating through a mechanism whereby the rapid oscillation of polar groups disrupts intramolecular interactions, consequently promoting the reorganization of protein secondary structures and partial unfolding of tertiary conformations [6]. Despite its proven efficacy protein modification, to our knowledge, microwave application to yuba processing is unexplored, except for the study using a 10 min microwave-assisted drying to enhance mechanical properties [7]. In contrast, how microwave treatment of soybeans alters protein aggregation and affects yuba quality remains unclear.

This study pioneers the application of short-duration microwave pretreatment to soybeans prior to soaking for subsequent yuba quality enhancement. It specifically examines how microwave treatment modulates soybean protein aggregation, thereby affecting soymilk properties and consequently influencing the mechanical, structural, and rehydration performance of yuba films. The proposed strategy significantly improved the mechanical strength and rehydration performance of yuba, while demonstrating exceptional industrial compatibility as a physical modification approach without the use of chemicals. These findings provide a theoretical foundation for the development of innovative, clean-label processing technologies in traditional soybean product manufacturing.

## 2. Materials and Methods

### 2.1. Materials and Reagents

Kedou 91 soybeans were obtained from the Keshan Branch of the Heilongjiang Academy of Agricultural Sciences (Qiqihar, China). Rhodamine B, BODIPY 505/515, and Calcofluor White were purchased from Shanghai Macklin Biochemical Co., Ltd. (Shanghai, China), Shanghai Hongye Biotechnology Co., Ltd. (Shanghai, China), and Sigma-Aldrich (Saint Louis, MO, USA), respectively. The BCA protein assay kit was sourced from Beijing Saiwen Innovation Biotechnology Co., Ltd. (Beijing, China). 5,5′-Dithiobis (2-nitrobenzoic acid) (DTNB) and Coomassie Brilliant Blue R-250 were obtained from Shanghai Maokang Biotechnology Co., Ltd. (Shanghai, China) and Sigma-Aldrich, respectively β-mercaptoethanol was purchased from Tianjin Guangfu Fine Chemical Research Institute (Tianjin, China). All other chemical reagents used were of analytical grade.

### 2.2. Yuba Film Preparation

#### 2.2.1. Preparation of Microwave-Pretreated Soybeans

Soybean raw material (2.0 kg, moisture content 8.20 ± 0.14%, protein content 30.44 ± 0.27% in dried basis, fat content 17.60 ± 0.81% in dried basis) was weighed and fed into the hopper of an XP-4KW laboratory-scale microwave expansion system (Guangzhou Weiyasi Microwave Equipment Co., Ltd., Guangzhou, China).

The microwave pretreatment was conducted in a cylindrical tunnel with a length of 1800 mm and an internal diameter of 140 mm. The material was conveyed at speeds of 60, 30, 20, and 15 mm/min, corresponding to microwave irradiation times of 30, 60, 90, and 120 s, resulting in outlet temperatures of 30, 40, 60, and 70 °C, respectively.

Microwave pretreatment was carried out in a resonant cavity operating at a frequency of 2450 ± 60 MHz with a fixed output power of 4 kW. After microwave treatment, the processed material was continuously discharged through a screw conveyor.

#### 2.2.2. Preparation of Soymilk

Soybean samples (160.0 g) prepared as described above were rinsed with deionized water and subsequently soaked in deionized water at a solid-to-liquid ratio of 1:5 (*w*/*v*) for 12 h in a water bath maintained at 25 °C ± 1 °C. After soaking, the soaking water was drained, and deionized water was added at a ratio of 1:8 (*w*/*v*) and the sample was ground using a soybean milk machine (JYL-Y912, Joyoung Co., Ltd., Harbin, China) at 35,000 rpm for 30 s, followed by nanoscale fine grinding with a colloid mill (JM-L50, Shanghai Kelau Machinery Equipment Co., Ltd., Shanghai, China) at 3000 rpm for 180 s. The resulting slurry was filtered through a 200-mesh sieve to obtain soymilk. A control sample was prepared using untreated soybeans following the same procedure.

#### 2.2.3. Formation of Yuba Films from Soymilk

Soymilk with a solid content of 7.0% ± 0.05% and pH 7.5, prepared as described in Section 2.2.2, was used for yuba formation. The soymilk was boiled for 3 min, then transferred into a stainless-steel pot (80.0 cm × 30.0 cm × 8.0 mm). The pot was placed directly in a water bath, with its base fully immersed, and maintained at 85 °C in a water bath to facilitate yuba film formation. Films were collected at 20 min intervals until no further film could be formed. The resulting yuba films were dried in an oven at 60 °C for 6 h to achieve a moisture content of 7.68 ± 0.19%. Control yuba films were prepared from soymilk using soybeans that did not undergo microwave pretreatment.

### 2.3. CLSM Analysis of Microstructure in Microwave-Pretreated Soybean Cells

Isolated soybean cells were prepared following Huyan et al., with modifications [8]. Briefly, soybeans were soaked in ice water overnight, manually dehulled, and the cotyledons were mixed with distilled water at a 1:3 (*w*/*v*) ratio in bottles, followed by heating at 121 °C for 10 min to promote cell dissociation. The resulting slurry was subjected to wet sieving using a stack of sieves with apertures ranging from 70 µm to 100 µm. The fraction that passed through the 100 µm sieve but was retained on the 70 µm sieve was collected as the intact cell isolate. The cells were resuspended in sterile phosphate-buffered saline (PBS, 0.01 mol/L) and stored at 5 °C until further analysis.

Cellular structure was examined by confocal microscopy according to Tao et al. [9]. Calcofluor White (100 μg/mL), Rhodamine B (10 μg/mL), and BODIPY 505/515 (1 μg/mL) were used to stain cell walls, protein bodies, and oil bodies, respectively. Equal volumes (1 mL each) of these dyes were thoroughly mixed, and 30 μL of the sample solution was combined with 30 μL of the dye mixture on a microscope slide. Imaging was performed using a 40× oil-immersion objective with excitation wavelengths of 405 nm, 543 nm, and 488 nm for Calcofluor White, Rhodamine B, and BODIPY 505/515, respectively. The resulting images were captured at a scanning density of 1024 × 1024 pixels and processed using LAS X 3.5.6 software (Leica Microsystems CMS GmbH, Wetzlar, Germany).

### 2.4. Physicochemical Properties Analysis of Soymilk

#### 2.4.1. Determination of Protein Concentration in Soymilk

Protein concentrations in soybean milk were determined using the BCA assay [10]. A standard curve was prepared using protein standards (0–2000 μg/mL) according to the kit protocol. The BCA working reagent was freshly prepared by mixing Reagent A and Reagent B at a 50:1 (*v*/*v*) ratio. Briefly, 20 μL of standard or diluted sample was mixed with 200 μL of the freshly prepared BCA working reagent in a 96-well plate, incubated at 37 °C for 30 min, then cooled at room temperature for 10 min, and the absorbance was measured at 562 nm (UV-5100, Shanghai Yuanxi Instrument Co., Ltd., Shanghai, China). Soy milk samples were diluted 50-fold to fall within the linear range of the assay.

#### 2.4.2. Extraction and Wet Weight Determination of Soybean Oil Bodies

The oil bodies were isolated according to the method described by Zhao et al. with minor modifications [11]. Five fresh soybean milk samples were combined with sucrose to achieve a final concentration of 20% (*w*/*w*), then centrifuged at 10,000 rpm or 30 min at 4 °C. The oil body layer was collected, and the liquid on the surface was absorbed with filter paper. The obtained layer was washed three times with deionized water to eliminate any free proteins, and any remaining moisture was absorbed.

#### 2.4.3. Analysis of Particle Size of Soymilk

The particle size distribution of fresh soymilk was analyzed using a Nano-ZS90 laser particle size analyzer (Malvern Panalytical Ltd., Malvern, UK) at 20 °C. Prior to measurement, the sample was diluted with deionized water to a protein concentration of 1 mg/mL and thoroughly mixed. Measurements were conducted in back-scattering geometry, with the scattered light collected at an angle of 173° using an avalanche photodiode.

#### 2.4.4. Determination of Stability Coefficient and Centrifugal Precipitation Rate in Soymilk

Soymilk stability coefficient was determined using a method adapted from Fan et al. [12]. In summary, the soybean milk was thoroughly shaken and diluted 1:15 (*v*/*v*) with distilled water, after which the initial absorbance (A_0_) was recorded at 785 nm (UV-5100, Shanghai Yuanxi Instrument Co., Ltd., Shanghai, China). Next, 10.0 g of the shaken soymilk was centrifuged at 3500 rpm for 15 min. A 0.5 mL sample was collected from the middle phase, diluted in the same manner, and its absorbance (A_1_) was measured in triplicate. The stability coefficient was determined using Equation (1), with higher values indicating better stability.

(1) $$\mathrm{Stability}\;\mathrm{coefficient}=\frac{A_{1}}{A_{0}}\times 100\%$$

In this equation, A_0_ refers to the absorbance of the soybean milk prior to centrifugation, while A_1_ indicates the absorbance of the whey phase after centrifugation. For the sedimentation test, 10.0 g of homogenized soybean milk was carefully weighed and centrifuged at 3500 rpm for 15 min. After centrifugation, the supernatant was removed, and the weight of the precipitate (m_1_) was accurately recorded. Each sample was measured three times. The sedimentation rate from centrifugation was determined using Equation (2), with lower values indicating greater stability.

(2) $$\mathrm{Centrifugal}\;\mathrm{sedimentation}\;\mathrm{rate}\;(\%)=\frac{m_{1}}{m_{0}}\times 100$$

where m_0_ is the mass of sample before centrifugation, g; m_1_ is the mass of sediment after centrifugation, g.

### 2.5. Physicochemical and Structural Characterization of Yuba

#### 2.5.1. Determination of the Mechanical Properties of Yuba

The mechanical properties of the membrane were evaluated according to a modified method from Zhang et al. [13]. Dried yuba strips (2 cm × 7 cm) were equilibrated at 25 °C and 90% RH for 24 h. Tensile strength (TS) and elongation at break (EAB) were measured using a texture analyzer (TA-TX PlusC, Xiamen Supertech Instruments Co., Ltd., Xiamen, China) equipped with an A/TG probe. The test settings were: 4 cm grip distance, 5 g trigger force, 1 mm/s pre-test and test speed, and 5 mm/s post-test speed. A minimum of five replicates was performed per sample.

The formula for calculating the TS of the yuba is given in Equation (3): F/STS (g/mm^2^)

(3) $$\mathrm{TS}\;(g/mm^{2})=F/S$$

where TS is the tensile strength (g/mm^2^), F is the force at break (g), and S is the cross-sectional area of the yuba (mm^2^).

The formula for calculating the EAB of the yuba is presented in Equation (4):

(4) $$EAB\;\left(\%\right)=(L_{1}-L_{0})/L_{0}\times 100$$

where EAB is the elongation at break (%), *L*_0_ is the initial length of yuba (cm), and *L*_1_ is the stretched length at fracture (cm).

#### 2.5.2. Determination of Rehydration Ratio of Yuba

The rehydration procedure was performed following the method of Bi et al., with minor modifications as described below [14]. The 1st, 3rd, and 4th dried yuba sheets prepared by the aforementioned method were selected for the experiment. Rehydration was conducted in deionized water at 30 °C. After the samples absorbed surface moisture using filter paper, they were weighed immediately. Eleven time points for sampling were established: 15 s, 30 s, 45 s, 1 min, 3 min, 5 min, 10 min, 15 min, 20 min, 25 min, and 30 min. The rehydration ratio was calculated using Equation (5):

(5) $$F_{f}\;=\;\frac{M_{f}}{M_{g}}$$

where F_f_ is the rehydration ratio; M_f_ is the mass of the yuba after rehydration, g; M_g_ is the mass of the yuba before rehydration, g.

#### 2.5.3. Yield Determination of Yuba

Yuba films were collected at 8 min intervals. Upon accumulation of three film layers, 100 mL of deionized water was immediately added to the soymilk, maintaining a solid content of 7.0% ± 0.05%, with rapid stirring. The harvest-and-replace cycle was repeated until visual inspection indicated cessation of film formation at the air-soymilk interface.

The yuba yield was calculated using Equation (6):

(6) Y (%) = m/(MC)×100

where m (g) was dry mass of the complete yuba batch; C (%) was the solid content of soymilk; M (g) was the soymilk mass.

#### 2.5.4. Determination of the Color of Yuba

Yuba is crushed into a fine powder and passed through a 50-mesh sieve for color measurement. A high-precision colorimeter is used to randomly measure the L*, a*, and b* values of the yuba powder at 5 points, and the average value is taken to represent the color of the yuba. The color of the standard white board is L* = 98.22, a* = −0.44, and b* = −0.42 (Shenzhen Linshang Technlolgy Co., Ltd. Shenzhen, China).

#### 2.5.5. Analysis of the Secondary Structure of Yuba Using FTIR Spectroscopy

For infrared spectroscopy analysis using the potassium bromide pellet method, 2.0 mg yuba (dry basis) powder was finely ground with 200 mg of solid potassium bromide in a mortar to create a consistent powder mixture, which was then pressed into pellets for testing (Perkin Elmer Inc., Norwalk, CT, USA). A Fourier transform infrared spectrometer was used for detection, with pure potassium bromide pellets serving as a blank control. The parameters for spectral acquisition were established as follows: a wavenumber range of 400–4000 cm^−1^, a resolution of 4.0 cm^−1^, and 32 scans were performed in transmission mode.

#### 2.5.6. Analysis of Protein Aggregation in Yuba by SDS-PAGE

SDS-PAGE was carried out according to Yang et al. with minor modifications [15]. Dried yuba was ground into a fine powder. Yuba powder was suspended in 8 mL of 8 mol/L urea, and 50 μL of β-mercaptoethanol was then added to 8 g of the powder. The mixture was incubated with gentle shaking at 25 °C for 24 h, then filtered. The resulting filtrate was collected and diluted to a final concentration of 0.3% (*w*/*v*, protein basis), which was subsequently mixed with an equal volume of loading buffer. After centrifugation, the supernatant was boiled for 5 min and cooled and prepared for SDS-PAGE analysis. Electrophoresis was performed using precast gels (12% separating gel, 5% stacking gel) at 120 V constant voltage until the tracking dye migrated 0.5–1.0 cm from the gel bottom. The gel was stained with Coomassie Brilliant Blue R-250 and destained with 10% methanol and acetic acid.

#### 2.5.7. Determination of Free Thiol and Disulfide Bond Contents in Yuba

Free and total sulfhydryl groups (-SH) in acetone-precipitated soymilk proteins were measured according to Guo et al. with modifications [16]. Soymilk (1 mL) was mixed with 9 mL of anhydrous acetone, stirred, and allowed to stand for 10 min. The mixture was centrifuged at 3000× *g* for 15 min, and the resulting precipitate was washed twice with 5 mL of acetone under the same centrifugal conditions. Finally, the residual solvent was evaporated using a stream of cold air. The precipitate was dissolved in 5 mL of Tris-glycine-EDTA buffer (10.4 g Tris, 6.9 g glycine, 1.2 g EDTA/L, pH 8.0) and Tris-glycine-EDTA-urea buffer (10.4 g Tris, 6.9 g glycine, 1.2 g EDTA, and 480 g urea/L, pH 8.0) to determine the content of sulfhydryl group on surface and total free sulfhydryl group, respectively.

For -SH determination, the precipitate obtained from 1 mL of pretreated soymilk was dissolved in 2 mL Tris-glycine-EDTA buffer and mixed with 0.02 mL Ellman’s reagent (4 mg/mL DTNB). The mixture was incubated at 25 °C for 5 min, and absorbance was measured at 412 nm.

For total sulfhydryl determination, 0.2 mL of the pretreated soymilk was mixed with 1 mL Tris-glycine-EDTA-urea buffer and 0.02 mL β-mercaptoethanol, followed by incubation at 25 °C for 1 h. Proteins were precipitated with 10 mL 12% (*w*/*v*) TCA for 1 h and centrifuged at 5000 rpm for 15 min; this step was repeated twice. The final pellet was redissolved in 3 mL Tris-glycine-EDTA buffer, reacted with 0.05 mL DTNB, and absorbance measured at 412 nm.

Determination of sulfhydryl content was performed using the following Equation (7):

(7) $$\mathrm{Sulfhydryl}\;\mathrm{group}\;(µ\mathrm{mol}/g\;\mathrm{protein})\;=\;(73.53\;\times {\;A}_{412}\;\times \;D)/C$$

where A_412_ is the absorbance at 412 nm, C is the total solid content of the sample, and D is the dilution factor after reagent addition.

The disulfide bond content was calculated using the following Equation (8):

(8) Disulfide bond (µmol/g protein) = (Reduced-SH−Non-reduced-SH)/2

-SH reduced and -SH non-reduced refer to the total sulfhydryl content under reducing and non-reducing conditions, respectively.

### 2.6. Statistical Analysis

The data are presented as means ± standard deviations from three separate experiments. To assess the differences between the means of various groups, post hoc testing following a one-way analysis of variance (ANOVA) was conducted, along with Duncan’s multiple range tests. The analysis was performed using SPSS Statistics 24.0 (IBM, New York, NY, USA).

## 3. Results

### 3.1. CLSM Analysis of Soybean Cells

**Figure 1:**
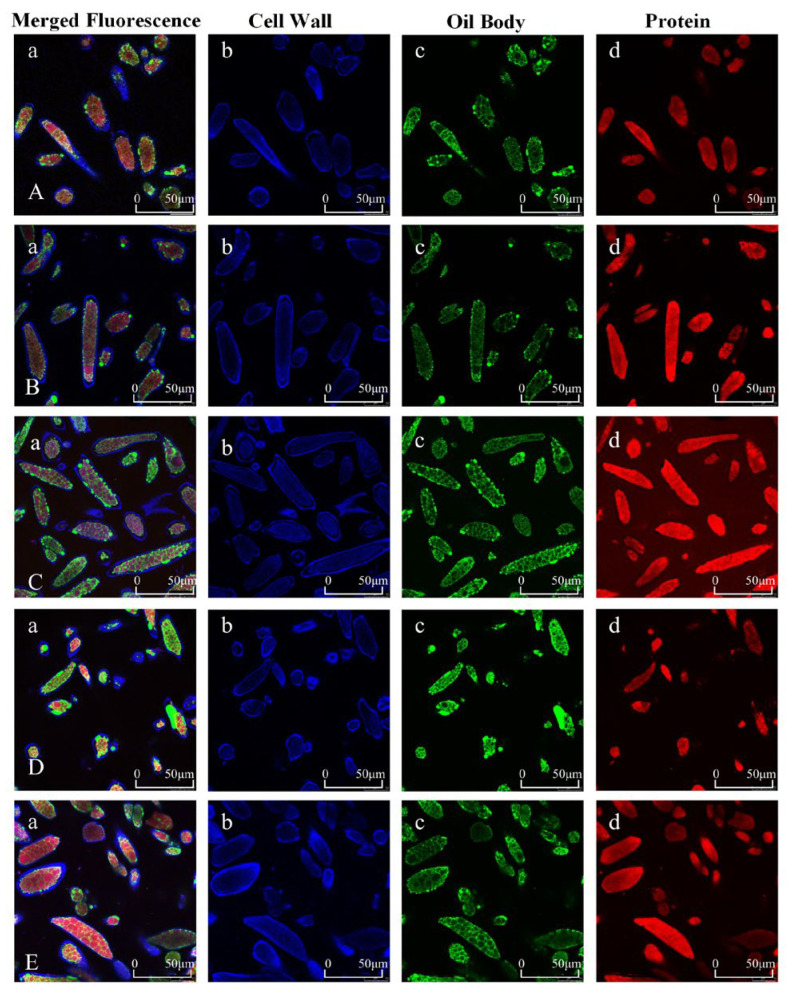
Confocal laser scanning microscopy images (**A**–**E**) of soybean cells after different microwave pretreatment durations (0, 30, 60, 90, and 120 s). (**a**) Merged fluorescence image of all three fluorophores. (**b**) Cell walls stained with Calcofluor White (blue). (**c**) Oil bodies stained with BODIPY 505/515 (green). (**d**) Proteins stained with Rhodamine B (red).

Confocal laser scanning microscopy (CLSM) was employed to observe the location and spatial distribution of protein (stained red) and oil bodies (green) within soybean cells, as illustrated in Figure 1. Oil bodies appeared as spherical structures densely packed between protein granules and cell walls. Microwave pretreatment disrupted the protein structure and altered the distribution of lipids and proteins. With increasing microwave duration, protein molecules gradually aggregated, forming a denser protein network that incorporated the oil bodies more effectively and resulted in a more organized spatial distribution of proteins and lipids. Moderate microwave treatment (30–90 s) promoted network formation and improved the uniformity of protein-lipid distribution, which is conducive to the mechanical strength of yuba. In contrast, prolonged treatment (120 s) led to excessive protein aggregation, disrupting the uniform distribution of proteins and lipids and potentially compromising the structural integrity of the protein network [17].

### 3.2. Effect of Soybean Microwave Pretreatment Duration on Soymilk Properties

#### 3.2.1. Oil Body Wet Weight and Soluble Protein Content of Soymilk Derived from Microwave-Pretreated Soybeans

The levels of oil bodies and soluble proteins in soymilk are closely related to yuba formation, as they affect the development and stability of the protein-lipid interfacial film during heating. As shown in Table 1, both protein content and oil body wet weight in soymilk exhibited an initial increase followed by a decrease as microwave pretreatment time extended. Specifically, oil body wet weight and protein content peaked at 30 s (58.10 ± 7.92 mg/mL) and 60 s group (40.41 ± 1.55 mg/mL), respectively, whereas both dropped to their minimums at 120 s group (30.99 ± 3.49 mg/mL and 31.15 ± 1.36 mg/mL), respectively. Moderate microwave treatment (30–90 s) facilitates the release of soybean oil bodies and enhances interactions among proteins, promoting the formation of a dense protein network and thereby improving the mechanical properties of yuba. In contrast, excessive microwave treatment (120 s) disturbed the protein-lipid distribution, resulting in a less stable protein network and reduced mechanical performance of yuba [3].

#### 3.2.2. Particle Size Analysis of Soymilk

**Figure 2:**
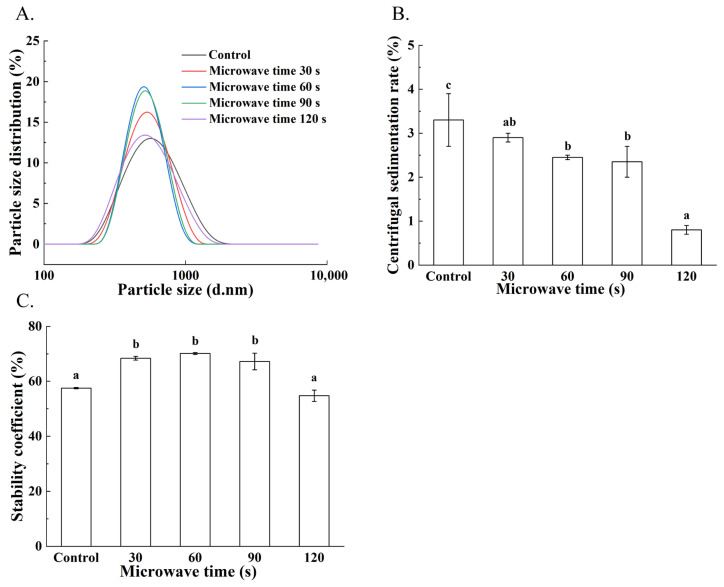
Properties of soymilk obtained from different microwave pretreatment durations of soybean. (**A**) Particle size distribution of soymilk, (**B**) Centrifugal sedimentation rate of soymilk, (**C**) Stability coefficient of soymilk. Different letters indicate significant differences among results (*p* < 0.05).

As shown in Table 2 and Figure 2A, microwave pretreatment significantly reduced the average particle size from 516.43 ± 5.63 nm (control) to smaller diameters, with a left-shift in the distribution profile indicating disaggregation of protein clusters. Microwave pretreatment significantly reduced the average particle size in treated samples compared with the control (516.43 ± 5.63 nm), as reflected by a leftward shift in the distribution profile, indicating disaggregation of protein clusters. The PDI values (0.07–0.16) further confirmed a relatively uniform particle distribution in the treated samples. This reduction can be attributed to microwave-induced unfolding of protein structures and cleavage of disulfide bonds, which in turn allows a rearrangement of proteins and oil bodies, leading to a more uniformly dispersed system. Smaller and more uniformly distributed particles in soymilk promote the assembly of a cohesive protein network during yuba formation, which is expected to enhance mechanical strength and rehydration properties [18].

#### 3.2.3. Stability and Centrifugal Sedimentation Rate of Soymilk

Soymilk stability was evaluated using the stability coefficient and centrifugal sedimentation rate. As shown in Figure 2B (stability coefficient) and Figure 2C (centrifugal sedimentation rate), the stability coefficient of soymilk increased with microwave pretreatment time, reaching a maximum of 70.12 ± 0.35%% at 60 s compared with 57.52 ± 0.25% in the control, and then decreased with prolonged treatment. In contrast, the centrifugal sedimentation rate showed an opposite trend, decreasing from 3.30 ± 0.60% in the control to lower values in the treated samples and reaching the minimum of 0.80 ± 0.10% at 120 s. This is due to appropriate microwave treatment, which exposes hydrophilic groups and reinforces the protein network, thereby increasing soymilk viscosity and reducing sedimentation [10,19]. Enhanced stability and reduced sedimentation of soymilk promote uniform protein-lipid distribution, supporting cohesive network formation in yuba.

### 3.3. Mechanical Properties of Yuba

**Figure 3:**
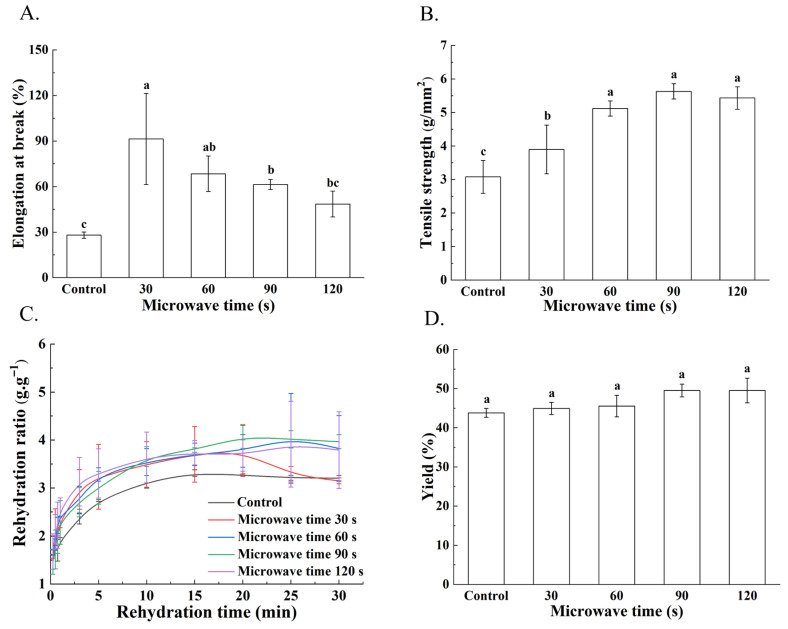
The variation in tensile strength (**A**), elongation at break (**B**), rehydration ratio (**C**), and yield (**D**) of yuba films as affected by microwave pretreatment duration applied to soybeans prior to soaking. Different letters indicate significant differences among results (*p* < 0.05).

The tensile strength (TS) and elongation at break (EAB) of yuba, which reflect its resistance to external forces and its extensibility, respectively, are presented in Figure 3A,B. TS increased progressively with microwave treatment, rising by 82.83% from 3.07 ± 0.49 g/mm^2^ in the control to 5.63 ± 0.23 g/mm^2^ at 60 s, which can be attributed to reduced hydrophobic group exposure and enhanced intermolecular crosslinking [20].

In contrast, EAB initially increased by 126.36% after 30 s of microwave pretreatment, from 27.99 ± 2.06% to 91.36 ± 29.94%, due to improved polymer chain mobility and network flexibility, but declined with prolonged treatment (120 s) as excessive crosslinking restricted molecular mobility [21]. Appropriate microwave treatment facilitated the formation of a more cohesive and balanced network structure, thereby enhancing the mechanical properties of yuba [22].

### 3.4. Rehydration Ratio of Yuba

Rehydration capacity is a key functional property of protein-lipid films, as yuba is typically stored and consumed in a dried form. Rehydration kinetics (Figure 3C) demonstrated that microwave-pretreated samples achieved higher water absorption rates within shorter timeframes. For instance, the 120 s group reached a rehydration ratio of 3.13 ± 0.50 g·g^−1^ in 3 min, a value matched by the control only after 15 min. Microwave treatment alters the protein structure, exposing rehydration groups and enhancing rehydration, whereas prolonged exposure overly compacts the network and may reduce rehydration. In addition, a cohesive protein network also improves yuba’s mechanical strength [23,24].

### 3.5. Yield of Yuba

The yield of yuba, a critical metric of process efficiency, is directly determined by the efficacy of protein network formation through denaturation, aggregation, and interfacial assembly. Microwave pretreatment had no statistically significant difference in yuba yield (*p* > 0.05); however, an increasing trend was observed, with the maximum yield reaching 49.53 ± 1.62% (Figure 3D). This trend can be attributed to structural modifications in the protein network, where microwave treatment disrupts the native spatial structure of proteins, promotes the formation of new cross-links, and alters the distribution of proteins and lipids, resulting in a stronger and more compact network that improves the overall quality of yuba [23]. Compared to conventional microwave processing, our method significantly reduces the treatment time and enables continuous soybean processing via a tunnel-type microwave processing equipment, collectively enhancing overall productivity.

### 3.6. Color Analysis of Yuba

Color parameters (Table 3) exhibited complex trends: L* (lightness) decreased initially then increased, reaching the highest value at 120 s (68.42 ± 0.52). Meanwhile, a* (redness) and b* (yellowness) increased initially before decreasing. These changes are attributed to Maillard reaction promotion under moderate microwave exposure, followed by pigment degradation or caramelization under excessive treatment.

### 3.7. FT-IR Analysis of Yuba

**Figure 4:**
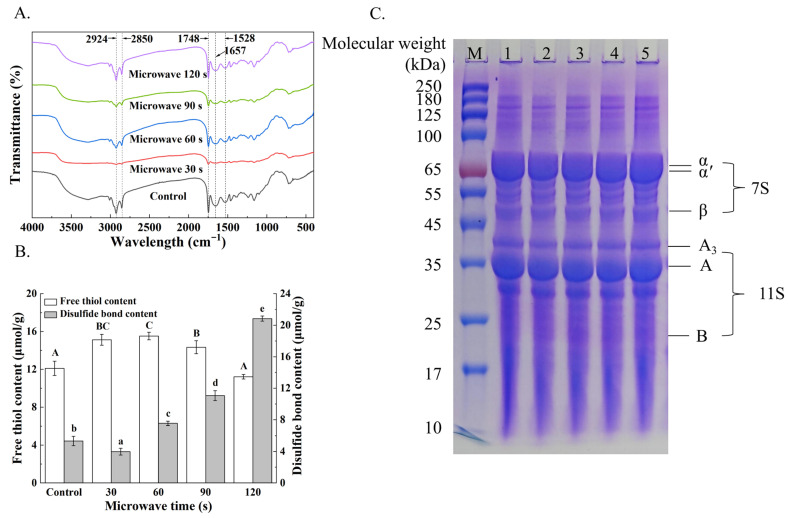
The variation in FTIR spectra (**A**), content of free sulfhydryl groups and disulfide bonds (**B**), and SDS-PAGE profile (**C**) of yuba films with different durations of microwave pretreatment applied to soybeans prior to soaking. Different letters in (**B**) indicate significant differences among results (*p* < 0.05).

FTIR spectroscopy was employed to characterize the structural changes in yuba proteins induced by microwave treatment. As shown in Figure 4A, the spectra exhibited similar overall molecular fingerprints across treatments. Reduced intensity in the 3200–3400 cm^−1^ region (O-H/N-H) indicated protein curling and reduced polar group exposure [25]. Persistent CH_2_ peaks (2924/2850 cm^−1^) suggested stable aliphatic chains, while amide I intensity reductions implied hydrogen bond disruption [26]. These findings provide direct evidence that microwave pretreatment alters protein conformation, and these conformational adjustments are likely responsible for the altered aggregation and intermolecular interactions that form a stronger and more cohesive protein network [27].

### 3.8. Determination of Protein Molecular Weight Distribution of Yuba

SDS-PAGE analysis revealed microwave-induced changes in protein aggregation and subunit composition in yuba. The two major soybean protein components, 11S glycinin and 7S β-conglycinin, play crucial roles in the network formation of yuba. As shown in Figure 4C, the electrophoretic profiles revealed that yuba is primarily composed of the α′, α, and β subunits of β-conglycinin (7S) along with the acidic (A) and basic (B) subunits of glycinin (11S). With extended microwave treatment of soybean raw material, a notable increase in prepared yuba was observed in the band intensities corresponding to the α′ and α subunits of 7S and the acidic subunit A of 11S. Collectively, these results suggest that microwave pretreatment promotes protein aggregation and intermolecular interactions in soymilk, as reflected by the enhanced intensities of specific 7S and 11S subunits, thereby strengthening the protein network and contributing to the improved mechanical properties and rehydration ratio of yuba [28].

### 3.9. Free Sulfhydryl Groups and Disulfide Bond Contents of Yuba

During the formation of yuba, heating the soybean milk exposes and accumulates free sulfhydryl groups (-SH), one of the most reactive functional groups in proteins, which are typically involved in protein oxidation and conformational stabilization. Therefore, free sulfhydryl groups can be used as an indicator of structural changes in yuba [5]. As shown in Figure 4B, short-term microwave treatment (≤60 s) resulted in an increase in free sulfhydryl (-SH) groups to 15.52 ± 0.39 μmol/g, corresponding to partial unfolding of proteins and exposure of free thiol groups. With extended microwave exposure (120 s), -SH content decreased to 11.21 ± 0.23 μmol/g, while disulfide bond content increased steadily to 20.83 ± 0.32 μmol/g. These observations demonstrate that microwave treatment induces oxidative cross-linking through the conversion of exposed -SH groups into disulfide bonds, thereby promoting the formation of a more compact and structurally stable protein network [29,30].

## 4. Conclusions

This study demonstrates that microwave pretreatment of soybeans prior to soaking effectively enhances the mechanical strength and rehydration properties of yuba. The 90 s pretreatment was identified as the optimal condition, producing the greatest improvements in elongation at break and rehydration capacity. This effect is attributed to microwave-induced alterations in protein conformation, which promote the redistribution of proteins and oil bodies and consequently enhance yuba quality. Molecular analysis revealed significant protein structural modifications, including enhanced crosslinking through disulfide bond formation. The pretreatment notably improved the mechanical properties, rehydration ratio, and overall quality of yuba products while maintaining processing yield. However, the precise molecular mechanisms driving these conformational changes and the industrial scalability of this approach require further investigation. These findings demonstrate that microwave pretreatment of soybeans prior to soaking serves as an innovative approach in yuba production, offering a promising technological advancement for the plant-based protein processing industry in enhancing product functionality and quality.

## Figures and Tables

**Table 1:** Protein content and wet weight of oil bodies in soymilk obtained from different microwave pretreatment durations of soybean.

Parameter	Microwave Time (s)				
	Control	30	60	90	120
Protein content (mg/mL) Wet weight of oil bodies (mg/mL)	36.14 ± 0.55 ^b^ 34.55 ± 8.42 ^a,b^	37.43 ± 0.79 ^b^ 58.10 ± 7.92 ^c^	40.41 ± 1.55 ^c^ 45.46 ± 5.33 ^b^	37.46 ± 0.87 ^b^ 37.65 ± 4.73 ^a,b^	31.15 ± 1.36 ^a^ 30.99 ± 3.49 ^a^

Different letters within the same row indicate that the means are significantly different according to one-way ANOVA analysis (*p* < 0.05).

**Table 2:** Particle size distribution of soymilk obtained from different microwave pretreatment durations of soybean.

Parameter	Microwave Time (s)				
	Control	30	60	90	120
Particle size (d.nm) PDI	516.43 ± 5.63 ^a^ 0.155	483.20 ± 8.46 ^b^ 0.070	487.53 ± 7.51 ^b^ 0.069	493.90 ± 11.16 ^b^ 0.089	488.87 ± 9.48 ^b^ 0.069

Different letters within the same row indicate that the means are significantly different according to one-way ANOVA analysis (*p* < 0.05).

**Table 3:** Color of Yuba Films formation from the soymilk obtained from different microwave pretreatment durations of soybean.

	Chromaticity Index	L*	a*	b*
Microwave Time (s)				
0 (Control)		65.99 ± 0.42 ^c^	3.66 ± 0.08 ^a^	22.54 ± 0.31 ^b^
30		68.37 ± 0.27 ^b^	3.51 ± 0.07 ^b^	22.46 ± 0.49 ^b^
60		66.86 ± 0.76 ^d^	3.70 ± 0.03 ^a^	23.09 ± 0.21 ^a^
90		67.85 ± 0.45 ^b^	2.85 ± 0.01 ^c^	22.60 ± 0.18 ^b^
120		69.42 ± 0.52 ^a^	2.72 ± 0.02 ^d^	21.76 ± 0.36 ^c^

Different letters within the same column indicate that the means are significantly different according to one-way ANOVA analysis (*p* < 0.05).

## Data Availability

The original contributions presented in this study are included in the article. Further inquiries can be directed to the corresponding authors.
